# Supplementary material for: Association of the Estimated Pulse Wave Velocity with Cardio-Vascular Disease Outcomes among Men and Women Aged 40–69 Years in the Korean Population: An 18-Year Follow-Up Report on the Ansung–Ansan Cohort in the Korean Genome Environment Study
Source: J Pers Med. 2022 Sep 30;12(10):1611. doi: 10.3390/jpm12101611 (PMC9605152; doi:10.3390/jpm12101611)
Supplement: Supplementary file 1 [file jpm-12-01611-s001.zip › jpm-1911746-supplementary.pdf]

Journal of Personalized Medicine

Supplementary Materials

Table S1. Optimal cut-off levels of ePWV for discrimination between patients with and without cardiovascular mortality and cardiovascular disease outcomes.

**Cardiovascular Mortality**	**AUC**	**Cut-off level of ePWV (m/s)**	**Sensitivity (%)**	**Specificity (%)**
At 120 months	0.815	10.08	71.90	79.38
At 144 months	0.817	8.87	89.51	59.95
At 168 months	0.830	9.70	77.57	74.78
At 192 months	0.817	9.68	74.99	75.27
At 216 months	0.825	9.11	85.59	66.50
Cardiovascular disease outcomes	AUC	Cut-off level of ePWV (m/s)	Sensitivity (%)	Specificity (%)
At 120 months	0.712	8.83	73.25	60.58
At 144 months	0.710	8.87	71.66	62.34
At 168 months	0.710	8.86	70.99	62.78
At 192 months	0.699	8.86	67.90	64.03
At 216 months	0.710	8.82	69.29	64.48

ePWV, estimated pulse wave velocity; AUC, area under the ROC curve.

Table S2. Hazard ratios for cardiovascular mortality and cardiovascular disease outcomes according to the quartiles of the estimated pulse wave velocity with second and third quartiles as references.

**Cardiovascular Mortality**	**Unadjusted HR (95% CI)**	**Model 1 [a] HR (95% CI)**	**Model 2 [b] HR (95% CI)**	**Model 3 [c] HR (95% CI)**
First quartile (4.52–7.38 m/s)	0.20 (0.06–0.70)	0.28 (0.08–1.00)	0.34 (0.10–1.21)	0.35 (0.10–1.25)
Second quartile (7.39–8.44 m/s)	REF	REF	REF	REF
Third quartile (8.45–9.89 m/s)	3.30 (1.85–5.90)	1.72 (0.90–3.29)	1.60 (0.82–3.12)	1.50 (0.77–2.94)
Fourth quartile (9.90–15.17 m/s)	11.6 (6.84–19.8)	3.76 (1.77–7.98)	3.32 (1.53–7.20)	3.06 (1.40–6.67)
Cardiovascular Disease Outcomes [d]	Unadjusted HR (95% CI)	Model 1 [a] HR (95% CI)	Model 2 [b] HR (95% CI)	Model 3 [c] HR (95% CI)
First quartile (4.52–7.38 m/s)	0.54 (0.43–0.68)	0.7 (0.55–0.9)	0.74 (0.58–0.94)	0.77 (0.6–0.98)
Second quartile (7.39–8.44 m/s)	REF	REF	REF	REF
Third quartile (8.45–9.89 m/s)	1.97 (1.66–2.34)	1.28 (1.05–1.57)	1.24 (1.01–1.52)	1.16 (0.94–1.42)
Fourth quartile (9.90–15.17 m/s)	3.21 (2.73–3.78)	1.33 (1.01–1.77)	1.26 (0.95–1.69)	1.15 (0.86–1.54)
Cardiovascular Mortality	Unadjusted HR (95% CI)	Model 1 [a] HR (95% CI)	Model 2 [b] HR (95% CI)	Model 3 [c] HR (95% CI)
First quartile (4.52–7.38 m/s)	0.06 (0.02–0.20)	0.17 (0.05–0.58)	0.21 (0.06–0.77)	0.23 (0.07–0.84)
Second quartile (7.39–8.44 m/s)	0.30 (0.17–0.54)	0.58 (0.30–1.11)	0.63 (0.32–1.22)	0.66 (0.34–1.30)
Third quartile (8.45–9.89 m/s)	REF	REF	REF	REF
Fourth quartile (9.90–15.17 m/s)	3.52 (2.54–4.87)	2.19 (1.45–3.31)	2.08 (1.36–3.19)	2.03 (1.32–3.13)
Cardiovascular Disease Outcomes [d]	Unadjusted HR (95% CI)	Model 1 [a] HR (95% CI)	Model 2 [b] HR (95% CI)	Model 3 [c] HR (95% CI)
First quartile (4.52–7.38 m/s)	0.27 (0.22–0.34)	0.55 (0.41–0.72)	0.6 (0.45–0.79)	0.66 (0.5–0.88)
Second quartile (7.39–8.44 m/s)	0.51 (0.43–0.6)	0.78 (0.64–0.95)	0.81 (0.66–0.99)	0.86 (0.7–1.06)
Third quartile (8.45–9.89 m/s)	REF	REF	REF	REF
Fourth quartile (9.90–15.17 m/s)	1.63 (1.43–1.86)	1.04 (0.87–1.25)	1.02 (0.85–1.23)	1.00 (0.82–1.20)

HR, hazard ratio; CI, confidence interval; REF, reference. [a] Model 1: Adjusted for age (per 10 years), sex, and systolic blood pressure (per 1 mmHg). [b] Model 2: Adjusted for variables included in Model 1 and smoking status, alcohol drinking status, physical activity (per 1 METs-hour/week), income level, and educational status. [c] Model 3: Adjusted for variables included in Model 2 and body mass index (per 1 kg/m2), waist circumference (per 1 cm), medical history (hypertension, diabetes mellitus, and dyslipidaemia), estimated glomerular filtration rate (per 1 mL/min/1.73 m2), fasting blood glucose level (per 1 mg/dL), total cholesterol level (per 1 mg/dL), and low-density lipoprotein cholesterol level (per 1 mg/dL). [d] Cardiovascular disease outcomes were defined as a composite of cardiovascular mortality, myocardial infarction, coronary artery disease, stroke, heart failure, and peripheral artery disease.

Table S3. Baseline characteristics according to sex.

Characteristics	Women (n = 5,118)	Men (n = 4,580)	p-Value
Age, mean (SD), year	52.5 (9.0)	51.6 (8.7)	<0.001
Body mass index, mean (SD), kg/m^2	24.9 (3.3)	24.2 (2.9)	<0.001
Waist circumference, mean (SD), cm	82.1 (9.7)	83.8 (7.7)	<0.001
Income level, n (%)			<0.001
≥Median	2108 (42.2)	2578 (56.9)	
Educational status, n (%)			<0.001
Lower than middle school	2285 (45.1)	927 (20.4)	
Middle school	1182 (23.3)	1027 (22.6)	
High school	1291 (25.5)	1639 (36.0)	
University and college	307 (6.1)	959 (21.1)	
Smoking status, n (%)			<0.001
Current smoker	192 (3.83)	2275 (50.0)	
Ex-smoker	66 (1.32)	1393 (30.6)	
Never-smoker	4754 (94.9)	886 (19.5)	
Alcohol drinking status, n (%)			<0.001
Current drinker	1305 (25.8)	3255 (71.5)	
Ex-drinker	155 (3.07)	456 (10.0)	
Never-drinker	3596 (71.1)	843 (18.5)	
Physical activity, mean (SD), METs-hour/week	167 (102)	178 (107)	<0.001
Systolic blood pressure, mean (SD), mmHg	124.0 (19.9)	125.1 (17.4)	0.003
Diastolic blood pressure, mean (SD), mmHg	80.2 (12.1)	83.2 (11.2)	<0.001
Medical history, n (%)			
Hypertension	838 (16.4)	580 (12.7)	<0.001
Diabetes mellitus	296 (5.8)	331 (7.2)	0.004
Dyslipidaemia	97 (1.9)	131 (2.9)	0.002
Chronic kidney disease	177 (3.5)	85 (1.9)	<0.001
Laboratory data, mean (SD)			
eGFR, mL/min/1.73 m^2	91.7 (20.9)	87.8 (19.5)	<0.001
Fasting blood glucose level, mg/dL	90.4 (20.8)	94.6 (24.7)	<0.001
Hemoglobin A1c level, %	5.8 (0.9)	5.8 (0.9)	0.103
Total cholesterol level, mg/dL	199.0 (36.8)	197.7 (36.7)	0.088
Triglyceride level, mg/dL	137.6 (92.3)	170.3 (124.1)	<0.001
HDL cholesterol level, mg/dL	51.1 (11.9)	47.9 (11.6)	<0.001
LDL cholesterol level, mg/dL	123.0 (31.5)	119.4 (129.4)	0.055

Data are presented as n (%) or means (SDs), as appropriate. SD, standard deviation; eGFR, estimated glomerular filtration rate; HDL, high-density lipoprotein; LDL, low-density lipoprotein.

Table S4. Reclassification of the predicted risk of cardiovascular events by the ePWV when added to the 10-year ASCVD risk model.

Cardiovascular events	NRIe	NRIne	Overall NRI [a]
Cardiovascular mortality	0.114 (0.049–0.187)	0.021 (0.031–0.011)	0.094 (0.032–0.162)
Cardiovascular disease outcomes [b]	0.167 (0.127–0.212)	0.063 (0.076–0.037)	0.105 (0.054–0.172)

ePWV, estimated pulse wave velocity; ASCVD, atherosclerotic cardiovascular disease; NRI, net reclassification index; NRIe, event NRI; NRIne, non-event NRI. [a] Overall NRI = NRIe-NRIne. [b] Cardiovascular disease outcomes were defined as a composite of cardiovascular mortality, myocardial infarction, coronary artery disease, stroke, heart failure, and peripheral artery disease.
